# Relation between Mid-Regional Pro-Adrenomedullin in Patients with Chronic Heart Failure and the Dose of Diuretics in 2-Year Follow-Up—Data from FAR NHL Registry

**DOI:** 10.3390/medicina58101477

**Published:** 2022-10-18

**Authors:** Monika Špinarová, Jindřich Špinar, Lenka Špinarová, Jan Krejčí, Monika Goldbergová-Pávková, Jiří Pařenica, Ondřej Ludka, Filip Málek, Petr Ošťádal, Klára Benešová, Jiří Jarkovský, Karel Lábr

**Affiliations:** 1 First Department of Internal Medicine—Cardioangiology, St. Anne’s University Hospital, Faculty of Medicine, Masaryk University, 625 00 Brno, Czech Republic; 2 Department of Pathological Physiology, Faculty of Medicine, Masaryk University, 625 00 Brno, Czech Republic; 3 Department of Internal Cardiology Medicine, Faculty Hospital Brno, Faculty of Medicine, Masaryk University, 625 00 Brno, Czech Republic; 4 Department of Internal Medicine, Geriatrics and Practical Medicine, Faculty Hospital Brno, Faculty of Medicine, Masaryk University, 625 00 Brno, Czech Republic; 5 Department of Cardiology, Na Homolce Hospital, 150 00 Prague, Czech Republic; 6 Institute of Biostatistics and Analyses, Faculty of Medicine, Masaryk University, 625 00 Brno, Czech Republic

**Keywords:** chronic heart failure, mid-regional pro-adrenomedullin, diuretics, furosemide, prognosis

## Abstract

*Background and Objectives*: The aim of this paper is to evaluate the impact of humoral substance mid-regional pro-adrenomedullin (MR-proADM) on the two-year survival of patients with chronic heart failure and relate it to the dosage of furosemide. *Materials and Methods*: The data is taken from the stable systolic heart failure (EF < 50%) FAR NHL registry (FARmacology and NeuroHumoraL activation). The primary endpoint at two-year follow-up was death, heart transplantation, or LVAD implantation. *Results*: A total of 1088 patients were enrolled in the FAR NHL registry; MR-proADM levels were available for 569 of them. The mean age was 65 years, and 81% were male. The aetiology of HF was ischemic heart disease in 53% and dilated cardiomyopathy in 41% of patients. The mean EF was 31 ± 9%. Statistically significant differences (*p* < 0.001) were obtained in several parameters: patients with higher MR-proADM levels were older, rated higher in NYHA class, suffered more often from lower limb oedema, and had more comorbidities such as hypertension, atrial fibrillation, diabetes, and renal impairment. MR-proADM level was related to furosemide dose. Patients taking higher doses of diuretics had higher MR-proADM levels. The mean MR-proADM level without furosemide (*n* = 122) was 0.62 (±0.55) nmol/L, with low dose (*n* = 113) 1–39 mg/day was 0.67 (±0.30) nmol/L, with mid dose (*n* = 202) 40–79 mg/day was 0.72 (±0.34) nmol/L, with high dose (*n* = 58) 80–119 mg/day was 0.85 (±0.40) nmol/L, and with maximum dose (*n* = 74) ≥120 mg/day was 1.07 (±0.76) nmol/L, *p* < 0.001. Patients with higher MR-proADM levels were more likely to achieve the primary endpoint at a two-year follow-up (*p* < 0.001) according to multivariant analysis. *Conclusions*: Elevated plasma MR-proADM levels in patients with chronic heart failure are associated with an increased risk of death and hospitalization. Higher MR-proADM levels in combination with increased use of loop diuretics reflect residual congestion and are associated with a higher risk of severe disease progression.

## 1. Introduction

Chronic heart failure (HF) is a clinical syndrome with a serious prognosis and increasing incidence. It is characterized by typical symptoms (e.g., breathlessness, ankle swelling, and fatigue) that may be accompanied by signs (e.g., elevated jugular venous pressure, pulmonary crackles, and peripheral oedema) [1].

Plasma concentrations of natriuretic peptides are recommended as initial diagnostic tests in patients with symptoms suggestive of HF in order to rule out the diagnosis [1]. They can help with guiding the therapy and also reflect the severity of the disease [2,3]. However, their informative value is still insufficient, because there are many other causes of elevated natriuretic peptides. Therefore, novel biomarkers, which could potentially predict the outcome of patients with chronic HF more accurately, are constantly under research.

Adrenomedullin (ADM) is a member of the calcitonin gene-related peptide (CGRP) superfamily [4]. It is synthesized mostly by endothelial and vascular smooth muscle cells. It contains 52 amino acids and is derived from a larger precursor (pre-proADM) by posttranslational processing, leaving two smaller biologically inactive fragments (the mid-regional part of proADM and the C-terminus of the molecule) [4]. ADM’s main function is vasodilatation, but it also increases renal blood flow and affects natriuresis and diuresis. ADM is also attributed to anti-inflammatory, anti-apoptotic, and proliferative effects, and it therefore appears to have protective properties in many pathological conditions [4,5]. ADM expression can be induced by various impulses; one of the main impulses is volume overload [6]. Furthermore, ADM interacts with the renin-angiotensin-aldosterone system and increases plasma renin activity [7].

ADM circulates in the plasma in very low concentrations in healthy humans; however, it rapidly increases in patients with heart failure [8]. Nevertheless, ADM routine measurement is not suitable for clinical routine diagnostics because of its instability, but mid-regional proADM is a stable and reliable surrogate for measurement markers of ADM release [9,10].

## 2. Materials and Methods

These data come from the FAR NHL registry (FARmacology and NeuroHumoraL activation), which is a database of patients with stable systolic heart failure (EF < 50%). Medical history data, and physical examination and biochemistry results including NT-proBNP were collected from seven departments with specialized heart failure care in three university hospitals in the Czech Republic between November 2014 and November 2015.

The primary endpoint, i.e., the two-year prognosis in terms of all-cause mortality, heart transplantation, and/or left ventricular assist device (LVAD) implantation, was evaluated up to November 2017. Patients were followed up prospectively at outpatient departments and mortality rates were verified in the National Death Certificate System. Monitored data on all patients were gathered at the end of the two-year follow-up. Eligible patients had to be treated for heart failure with non-preserved ejection fraction (EF < 50%) and to be stable for at least one month with an optimal pharmacotherapy treatment, as recommended in ESC guidelines for chronic heart failure. Exclusion criteria were not signing the informed consent, signs and symptoms of acute decompensation of heart failure, and conditions other than heart failure that would certainly limit the mid-term prognosis of patients (e.g., advanced stage of cancer, severe dementia, and others). Patients with an orthotopic heart transplant (OHT) or with a left ventricle assistance device (LVAD) implantation in the baseline were not included.

The study was conducted according to the guidelines of the Declaration of Helsinki and approved by the Ethics Committee of Brno University Hospital, Czech Republic (protocol code 02-221014/EK, date of approval 22 October 2014).

### 2.1. Study Population

In the whole FAR NHL registry, a total amount of 1088 patients were included. The level of MR-proADM was available in 569 of them. The mean age was 65 years, 80.7% were male, the aetiology of chronic HF was ischemic heart disease in 53.3%, and dilated cardiomyopathy in 40.5% and 6.2% were classified as other. Mean EF was 31 + 9%. The most common comorbidities were hypertension (64.1%) and diabetes mellitus (40.2%). Beta-blockers were administered to 93.1% of patients, drugs blocking the renin-angiotensin system (ACE inhibitors and/or ARBs) were administered to 88.4% of patients, and diuretics were administered to 88.0% of patients. The most prescribed diuretic was furosemide, which was given to a total of 78.6% of patients alone or in combination, followed by spironolactone or eplerenone, which was prescribed to 64.1% of patients, and hydrochlorothiazide 12.8%. Patients were classified as NYHA I at 13.7%, NYHA II at 66.3%, and NYHA III-IV at 20.0%. The baseline characteristic of the cohort is displayed in Table 1.

### 2.2. Statistical Methods

Standard descriptive statistics were applied in the analysis; continuous variables were described by mean ± SD or in the case of laboratory results by median and interquartile range, whereas categorical variables were characterized by absolute and relative frequencies. Statistical significance of differences among groups of patients was analysed using the Mann–Whitney test or the Kruskal–Wallis test for continuous variables and Fisher’s exact test for categorical variables.

The predictive power of MR-proADM for the combined endpoint was analysed using ROC analysis and described by AUC, its confidence interval, and statistical significance. The optimal cut-off was selected by maximization of the Youden index. Time to combined endpoint was visualized using the Kaplan–Meier methodology and the computation of the proportion of surviving patients and its 95% confidence interval in a given time point was also based on Kaplan–Meier estimates. Statistical significance of differences in time to the event among groups of patients was evaluated using the log-rank test. Parameters were assessed using univariable logistic regression models. Significant factors (*p* < 0.05) were entered into a multivariable logistic regression model with a backward stepwise algorithm for the selection of independent predictors of the primary endpoint. The analysis was performed using SPSS version 26.0 (IBM Corporation, Armonk, NY, USA, 2019).

### 2.3. Laboratory Data

All subjects underwent a blood test to determine the standard biochemical parameters, markers of liver and renal functions and NT-proBNP levels.

The plasma samples were collected, directly frozen, and stored at −80 °C. Biomarker of cardiovascular load—MR-proADM—was detected using the homogeneous sandwich fluor immunoassay on the automated KRYPTOR (B·R·A·H·M·S AG, Hennigsdorf/Berlin, Germany). The B·R·A·H·M·S KRYPTOR^®^ is a fully automated homogeneous random-access platform. The system uses the time-resolved amplified cryptate emission (TRACE) technology.

The detection limit of the kit B·R·A·H·M·S MR-proADM KRYPTOR (Thermo Scientific, Waltham, MA, USA) was 0.05 nmol/L, and the direct measurement range was 0.05–10 nmol/L, enhanced with the use of automatic dilution up to 100 nmol/L. The functional assay sensitivity (inter-assay precision of 20% CV) has been assessed as being 0.25 nmol/L. In the concentration range of >2–6 nmol/L was the intra-assay of <2% CV, and the inter-assay of ≤10% CV.

## 3. Results

Patients without a primary endpoint were assigned to group A (479 pts), and those with the primary endpoint to group B (86 pts). A total of 10 patients were LVAD implanted, 16 patients underwent heart transplantation and 66 patients died. Some of the patients from group B reached more than one endpoint in general. There were statistically significant differences between the groups in the levels of MR-proADM: group A median 0.62 nmol/L (0.48; 0.79) vs. group B 0.88 nmol/L (0.67; 1.21) (*p* < 0.001). The statistically significant difference (*p* < 0.001) was obtained in these parameters according to the level of MR-proADM: patients with a higher level of MR-proADM were older, evaluated by higher NYHA class, suffer more often from swollen lower limbs, and had more comorbidities such as hypertension, atrial fibrillation, diabetes mellitus, and renal dysfunction. The complete results are displayed in Table 2.

**Figure 1:**
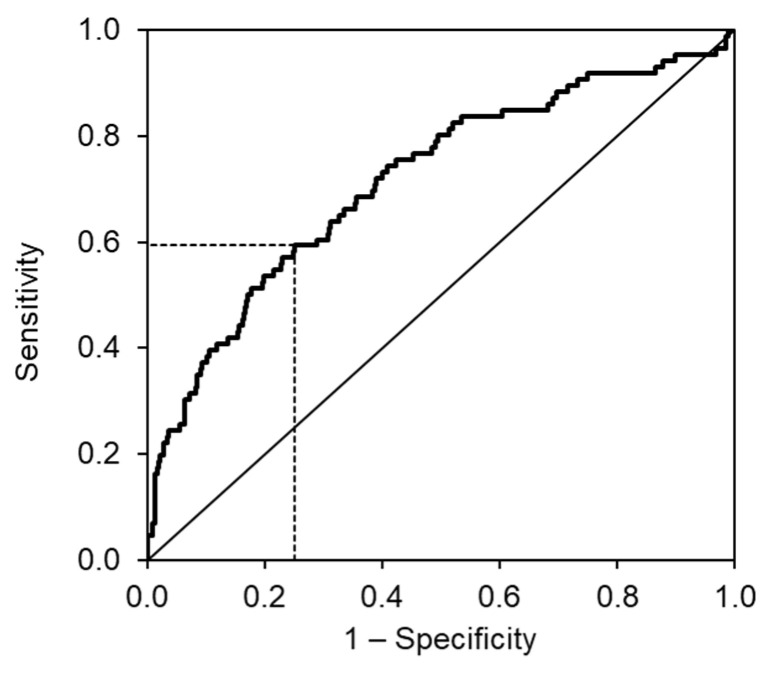
ROC curves for prediction of the primary endpoint (i.e., the two-year prognosis in terms of all-cause mortality, heart transplantation, LVAD implantation).

The cut-off value for MR-proADM was set to 0.79 nmol/L with 59.3% sensitivity and 74.9% specificity to predict primary outcome in the two-years follow-up (AUC = 0.716 (95% CI: 0.653–0.779), *p* < 0.001). Multivariable analysis has revealed that the dose of furosemide and MR-proADM are independent predictors of the primary endpoint. (Table 3). Although there is a correlation between MRproADM and furosemide dose (Spearman’s rank correlation coefficient = 0.318), the inclusion of both parameters in the model significantly improves the predictive ability of the model (Table 4). ROC curves for MR-proADM are displayed in Figure 1.

**Figure 2:**
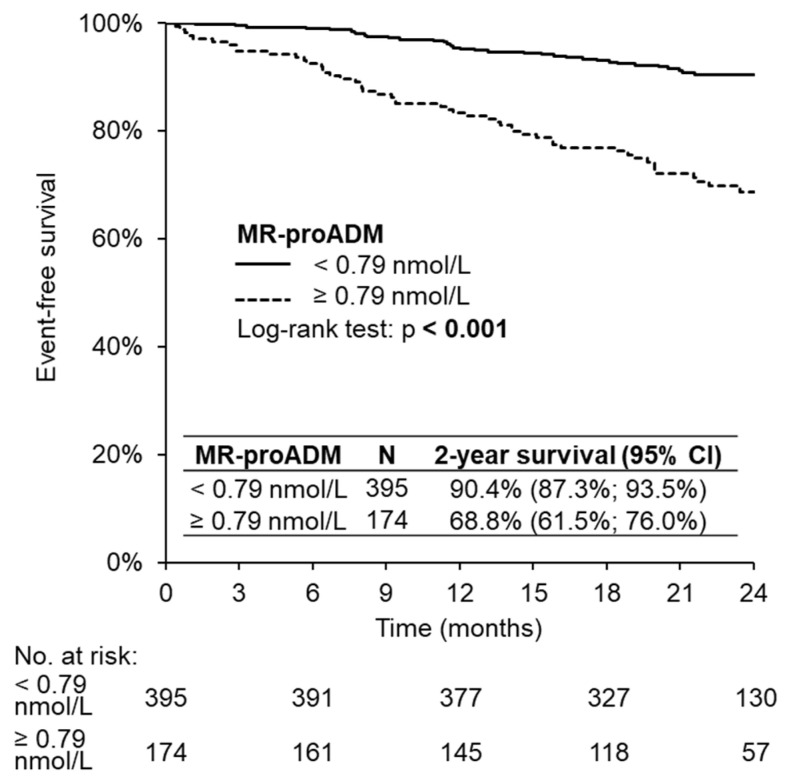
Two-year event-free survival.

According to the primary endpoint, patients with a higher level of MR-proADM reached the primary endpoint in two years of follow-up more often, which was statistically significant, *p* < 0.001 (Figure 2).

**Figure 3:**
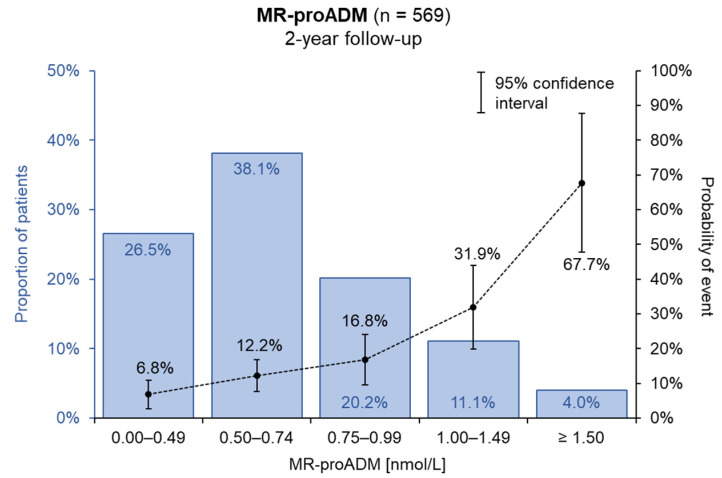
Distribution of MR-proADM level.

The distribution of MR-proADM levels in patients from our database during the two-year occurrence of the combined endpoint is displayed in Figure 3. Patients with a higher level of MR-proADM reveal a statistically significant worse prognosis in two years follow-up (*p* < 0.001).

The level of the MR-proADM was also related to the dose of furosemide. We have discovered that patients taking higher doses of diuretics are having a statistically significant higher level of MR-proADM. The mean level of MR-proADM in patients with no furosemide (*n* = 122) was 0.62 (±0.55) nmol/L, compared to patients with the maximum dose furosemide (*n* = 74) ≥ 120 mg/day where the mean level of MR-proADM was 1.07 (±0.76) nmol/L, *p* < 0.001. The level of MR-proADM was gradually increased according to the dosage of furosemide as it is displayed in Table 5.

## 4. Discussion

Mid-regional pro-adrenomedullin has already proved its prognostic value in several studies. According to the BACH study, MR-proADM had superior accuracy for predicting 90-day all-cause mortality in patients presenting with acute HF compared with BNP. MR-proADM was the biggest contributor to predictive performance [9].

Haehling et al. evaluated MR-proADM in 501 patients with chronic heart failure and revealed that plasma levels of MR-proADM correlate with the severity of heart failure, and are associated with a higher incidence of fatal events in one year of follow-up. The plasma level of MR-proADM in this study cohort was between 0.11 to 3.30 nmol/L, with a median of 0.64 nmol/L (IQR 0.49–0.87 nmol/L) [11]. The evaluation of MR-proADM provided independent information on prognosis in patients with chronic HF regardless of an assessment of NT-proBNP, as it is recommended in current European and North American guidelines for HF [1,11,12].

In our study, we have also proved the prognostic value of MR-proADM in 24 months of follow-up and our cut-off value corresponds to the value of Haeling. Our results also reveal a strong correlation between the level of MR-proADM and the severity of the disease. Our previous studies have also shown that the predictive value of MR-proADM is strongest in patients with low comorbidities according to the AHEAD score. AHEAD is a simple scoring system based on the analysis of comorbidities; A= atrial fibrillation, H = hemoglobin, E = elderly, A = abnormal renal function, and D = diabetes mellitus. The predictive value of MR-proADM declined with a higher level of AHEAD [10,13].

Many studies have shown that the main reason for rehospitalization for heart failure is related to pulmonary congestion [14,15,16]. The treatment of these patients is based on reducing the volume overload to alleviate the patient’s symptoms, improve exercise capacity, and reduce heart failure hospitalizations. Loop diuretics are recommended for these indications [1]. However, achieving complete decongestion is sometimes challenging, and in many patients, the congestion persists also after energetic diuretic therapy. Recent studies have discovered that reaching a level of decongestion that leads to relieving the symptoms of patients with chronic HF might still be unsatisfactory. Data from these studies have discovered that there are essential differences between the level of congestion that relieves symptoms and that improves the prognosis [17,18]. The level of congestion that persists after the initial treatment with diuretics is described as residual congestion. This congestion is not the cause of the patient’s symptoms but has a negative effect on the long-term prognosis of the patient [19]. The methods for detection of this state are still unsatisfactory and new possibilities need to be discovered to improve the current diagnostic algorithm for chronic systolic heart failure.

ADM could be one of these markers due to its pathophysiological properties. ADM levels rapidly decrease after treatment with diuretics. Persistent higher levels of ADM are associated with serious residual congestion and also a more severe prognosis. It could be suggested that patients who require higher doses of diuretics to manage their congestion are those in a more advanced condition of the disease, than those who require lower doses of loop diuretics based on their plasma level of MR-proADM. It can be concluded that higher levels of MR-proADM combined with increased use of loop diuretics are associated with a higher risk of serious progress of the disease. It may help to detect patients that are inadequately decongested or are resistant to diuretic therapy to re-evaluate their treatment strategy, such as changing to another loop diuretic, using a combination of diuretics, or using some of the elimination methods [20,21]. Otherwise, low levels of MR-proADM may help identify patients with lower residual congestion whose dose of diuretic appears to be adequate. Therefore, an improved evaluation of the residual congestion could be of significant importance in finding better and more personalized therapies for patients with chronic HF.

Elevated plasma levels of MR-proADM in patients with chronic HF reflect residual congestion and are associated with an increased risk of mortality and hospitalization.

### Limitations

Our study has several limitations. The FAR NHL study population is a Czech multicentric cohort which might not resemble the heterogeneity of all patient populations. Only patients with mid-range and reduced ejection fractions were evaluated, and patients with heart failure with preserved ejection fractions were not included in the study. Basic laboratory measurements including NT-proBNP were not performed in a core laboratory and may be subject to between-laboratory variability. We measured the stable part of the ADM precursor peptide, mid-regional pro-ADM (MR-proADM), however, the level of biologically active amidated ADM (bio-ADM) might be more accurate in the assessment of the endogenous level of ADM [22].

## 5. Conclusions

Adrenomedullin is a novel biomarker that is released as a counteracting response to volume overload. Mid-regional proADM is a stable and reliable surrogate for measurement markers of ADM release.

MR-proADM also appears as a predictor of a more serious prognosis in two years follow-up. Higher levels of MR-proADM combined with increased use of loop diuretics are associated with a higher risk of serious progress of the disease and may refer to a reassessment of the treatment strategy.

## Figures and Tables

**Table 1:** Baseline characteristics of patients (*N* = 569).

Parameter	Description *
**Basic characteristics**	
Sex—male	459 (80.7%)
Age (years)	65 ± 12
BMI	29 ± 5
SBP (mmHg)	127 ± 16
DBP (mmHg)	80 ± 10
Heart rate (min^−1^)	74 ± 13
LV EF (%)	31 ± 9
Ischemic aetiology of HF	303 (53.3%)
Hypertension	365 (64.1%)
Diabetes mellitus	229 (40.2%)
COPD	82 (14.4%)
Chronic lower extremity ischemia	56 (9.8%)
Smoking	
Non-smoker	326 (57.3%)
Smoker	56 (9.8%)
Ex-smoker	187 (32.9%)
NYHA classification	
1	78 (13.7%)
2	377 (66.3%)
3 + 4	114 (20.0%)
**Laboratory results**	
MR-proADM (nmol/L)	0.64 (0.49; 0.84)
NT-proBNP (ng/L)	662 (216; 1 838)
Haemoglobin (g/L)	144 (133; 153)
Natrium (mmol/L)	141 (139; 143)
Urea (mmol/L)	6.1 (4.9; 8.2)
Uric acid (µmol/L)	399 (339; 467)
Creatinine (μmol/L)	96 (83; 116)
eGFR (mL/min/1.73 m^2^)	69 (51; 84)
**Medication**	
ACEI/ARB	503 (88.4%)
Beta-blockers	530 (93.1%)
Furosemide	447 (78.6%)
Hydrochlorothiazide	73 (12.8%)
Spironolactone/eplerenone	365 (64.1%)

* Categorical parameters are described by absolute and relative frequencies. Continuous parameters are described by mean and standard deviation, in the case of laboratory results by the median and interquartile range (Q1; Q3). ACE, angiotensin-converting enzyme; ARB, angiotensin receptor blocker; BMI, body mass index; COPD, chronic obstructive pulmonary disease; DBP, diastolic blood pressure; eGFR, estimated glomerular filtration rate; HF, heart failure; LV EF, left ventricular ejection fraction; MR-proADM, mid-regional pro-adrenomedullin; NT-proBNP, N-terminal pro-B-type natriuretic peptide; NYHA, New York Heart Association; SBP, systolic blood pressure.

**Table 2:** Levels of MR-proADM.

Parameter	Category	*N*	Level of MR-proADM (nmol/L)			*p*
			Mean (±SD)	Median (25th Percentile; 75th Percentile)	OR (95% CI)	
Sex	Men	459	0.73 (±0.46)	0.64 (0.48; 0.83)	1.16 (0.64–2.11)	NS
	Women	110	0.80 (±0.58)	0.66 (0.53; 0.89)		
Age	<65 years	266	0.58 (±0.25)	0.53 (0.42; 0.68)	1.68 (1.05–2.70)	**<0.001**
	≥65 years	303	0.89 (±0.58)	0.75 (0.60; 0.98)		
BMI	<30	340	0.72 (±0.46)	0.63 (0.49; 0.82)	1.07 (0.67–1.71)	NS
	≥30	225	0.78 (±0.52)	0.67 (0.50; 0.86)		
LV EF	<40	441	0.76 (±0.53)	0.66 (0.49; 0.85)	0.51 (0.27–0.98)	NS
	≥40	128	0.67 (±0.28)	0.60 (0.49; 0.78)		
NYHA	1	78	0.54 (±0.22)	0.49 (0.40; 0.67)		**<0.001**
	2	377	0.77 (±0.50)	0.66 (0.53; 0.87)	2.70 (0.94–7.72)	
	3 + 4	114	0.80 (±0.54)	0.68 (0.51; 0.97)	7.86 (2.66–23.23)	
Ischemic aetiology	No	266	0.70 (±0.44)	0.60 (0.47; 0.80)	1.07 (0.67–1.69)	**0.013**
	Yes	303	0.78 (±0.51)	0.68 (0.54; 0.89)		
Hypertension	No	204	0.64 (±0.30)	0.61 (0.45; 0.77)	1.44 (0.87–2.37)	**<0.001**
	Yes	365	0.80 (±0.55)	0.67 (0.53; 0.91)		
Atrial fibrillation	No	373	0.65 (±0.32)	0.58 (0.47; 0.76)	1.02 (0.63–1.66)	**<0.001**
	Yes	196	0.92 (±0.67)	0.76 (0.62; 1.02)		
Diabetes mellitus	No	340	0.66 (±0.37)	0.60 (0.46; 0.78)	1.60 (1.01–2.53)	**<0.001**
	Yes	229	0.86 (±0.60)	0.71 (0.57; 0.98)		
Swollen lower limb	No	477	0.69 (±0.35)	0.62 (0.48; 0.80)	2.03 (1.17–3.50)	**<0.001**
	Yes	92	1.01 (±0.86)	0.78 (0.63; 1.08)		
eGFR [mL/min/1.73 m^2^]	<60	200	1.03 (±0.67)	0.84 (0.68; 1.17)	0.54 (0.34–0.86)	**<0.001**
	≥60	369	0.59 (±0.22)	0.55 (0.45; 0.69)		

*p*-value of Mann–Whitney U test or Kruskal–Wallis test is shown with Bonferroni correction applied. CI, confidence interval; OR, odds ratio; Ref., reference category. BMI, body mass index; eGFR, estimated glomerular filtration rate; HF, heart failure; LV EF, left ventricular ejection fraction; NS, not significant, NYHA, New York Heart Association.

**Table 3:** Multivariable logistic regression model of the primary endpoint (i.e., the two-year prognosis in terms of all-cause mortality, heart transplantation, LVAD implantation).

Parameter	Category	OR (95% CI)	*p*-Value
Dose of furosemide [mg/day]	1–39 (ref. 0)	0.79 (0.27–2.26)	0.656
	40–79 (ref. 0)	1.58 (0.68–3.67)	0.291
	80–119 (ref. 0)	2.76 (1.04–7.32)	**0.042**
	≥120 (ref. 0)	6.03 (2.46–14.79)	**<0.001**
MR-proADM	Increase of 0.1 nmol/L	1.14 (1.07–1.21)	**<0.001**

AUC = 0.778 (95% CI: 0.723–0.833; *p* < 0.001); sensitivity 72.1%, specificity 74.7%. AUC, the area under the curve; CI, confidence interval; OR, odds ratio; Ref., reference category.

**Table 4:** Two-year probability of primary endpoint based on a multivariable logistic regression model.

		MR-proADM [nmol/L]				
		0.00–0.49	0.50–0.74	0.75–0.99	1.00–1.49	≥1.50
**Dose of furosemide [mg/day]**	**0**	4.8%	7.1%	9.9%	18.6%	low N
	**1–39**	3.5%	5.1%	7.2%	13.9%	low N
	**40–79**	6.9%	10.1%	13.9%	25.1%	46.0%
	**80–119**	10.8%	15.5%	20.8%	35.4%	58.2%
	**120+**	21.7%	29.4%	37.4%	55.4%	76.0%

**Table 5:** Dose of diuretics according to the level of MR-proADM.

Parameter	Category	*N*	Level of MR-proADM (nmol/L)		*p*
			Mean Level of (±SD)	Median (25th Percentile; 75th Percentile)	
Dose of furosemide	0 mg/day	122	0.62 (±0.55)	0.55 (0.46; 0.67)	**<0.001**
	1–39 mg/day	113	0.67 (±0.30)	0.60 (0.44; 0.82)	
	40–79 mg/day	202	0.72 (±0.34)	0.67 (0.51; 0.81)	
	80–119 mg/day	58	0.85 (±0.40)	0.74 (0.58; 0.99)	
	≥120 mg/day	74	1.07 (±0.76)	0.82 (0.61; 1.23)	

The *p*-value of the Kruskal–Wallis test is shown with Bonferroni correction applied. Spearman’s rank correlation coefficient: ρ = 0.329, *p* <0.001.

## Data Availability

Data is available on request due to privacy and ethical restrictions. The data presented in this study are available on request from the corresponding author.
